# STAT5 Is an Ambivalent Regulator of Neutrophil Homeostasis

**DOI:** 10.1371/journal.pone.0000727

**Published:** 2007-08-15

**Authors:** Laurence Fiévez, Christophe Desmet, Emmanuelle Henry, Bernard Pajak, Silke Hegenbarth, Virginie Garzé, Françoise Bex, Fabrice Jaspar, Philippe Boutet, Laurent Gillet, Alain Vanderplasschen, Percy A. Knolle, Oberdan Leo, Muriel Moser, Pierre Lekeux, Fabrice Bureau

**Affiliations:** 1 Laboratory of Cellular and Molecular Physiology, GIGA-Research, University of Liège, Liège, Belgium; 2 Laboratory of Animal Physiology, Institute of Molecular Biology and Medicine, Université Libre de Bruxelles, Gosselies, Belgium; 3 Institute for Molecular Medicine and Experimental Immunology, University of Bonn, Bonn, Germany; 4 Laboratory of Microbiology, Institute for Microbiological Research J-M Wiame, Université Libre de Bruxelles, Brussels, Belgium; 5 Laboratory of Immunology and Vaccinology, Faculty of Veterinary Medicine, University of Liège, Liège, Belgium; Oklahoma Medical Research Foundation, United States of America

## Abstract

**Background:**

Although STAT5 promotes survival of hematopoietic progenitors, STAT5^−/−^ mice develop mild neutrophilia.

**Methodology/Principal findings:**

Here, we show that in STAT5^−/−^ mice, liver endothelial cells (LECs) autonomously secrete high amounts of G-CSF, allowing myeloid progenitors to overcompensate for their intrinsic survival defect. However, when injected with pro-inflammatory cytokines, mutant mice cannot further increase neutrophil production, display a severe deficiency in peripheral neutrophil survival, and are therefore unable to maintain neutrophil homeostasis. In wild-type mice, inflammatory stimulation induces rapid STAT5 degradation in LECs, G-CSF production by LECs and other cell types, and then sustained mobilization and expansion of long-lived neutrophils.

**Conclusion:**

We conclude that STAT5 is an ambivalent factor. In cells of the granulocytic lineage, it exerts an antiapoptotic function that is required for maintenance of neutrophil homeostasis, especially during the inflammatory response. In LECs, STAT5 negatively regulates granulopoiesis by directly or indirectly repressing G-CSF expression. Removal of this STAT5-imposed brake contributes to induction of emergency granulopoiesis.

## Introduction

Neutrophils represent 60% of the total circulating leukocytes in humans, and form the first line of host defence against invading microorganisms. Under physiological conditions, neutrophils undergo constitutive apoptosis within 6–18 h of entry into the bloodstream [1]. Therefore, 10^11^ neutrophils need to be produced daily in the bone marrow to maintain homeostasis [2]. In response to pathogen infection, neutrophils quickly migrate into peripheral tissues where they amplify the inflammatory response through the release of cytokines such as TNF-α [3]. While participating in the inflammatory process, neutrophils kill pathogens using several microbicidal mechanisms, including phagocytosis and production of reactive oxygen intermediates [3], [4]. The inflammatory response is accompanied by an expansion of the peripheral neutrophil pool that results from increased neutrophil survival and enhanced neutrophil production, a process known as emergency granulopoiesis [1], [5], [6].

The current model of hematopoiesis proposes that early in their differentiation, long-term hematopoietic stem cells (HSCs) lose their capacity for self-renewal, differentiating first into short-term HSCs and subsequently to multipotent progenitors (MPPs) [7]. MPPs generate common myeloid progenitors (CMPs) that are able to differentiate into the erythromyeloid lineages, and common lymphoid progenitors that produce B, T, and natural killer cells. CMPs may form two more restricted cell types, the granulocyte-macrophage progenitors (GMPs) and the megakaryocyte-erythrocyte progenitors. Neutrophils develop from GMPs through the myeloblast, the promyelocyte, the myelocyte, and the metamyelocyte stages.

G-CSF is recognized as the primary extracellular regulator of both basal and emergency granulopoiesis [8]–[11]. The importance of G-CSF to in vivo granulopoiesis was demonstrated in mice carrying a homozygous null mutation for G-CSF or G-CSF receptor. These mice had 12–30% of normal circulating neutrophils and a corresponding decrease in granulocytic precursors in their bone marrow [10], [11]. Multiple actions of G-CSF have been described that may contribute to the neutrophilic response. First, G-CSF stimulates proliferation and terminal differentiation of granulocytic precursors [12]. Second, it reduces the average transit time through the granulocytic compartment [12], [13]. Third, it regulates neutrophil trafficking from the bone marrow to the blood [14]. Fourth, it stimulates a broad range of mature neutrophil functions [15]. Finally, G-CSF prolongs neutrophil survival [16].

Most biological functions of G-CSF are mediated through STAT1, STAT3, and STAT5 [17]–[19]. However, a requirement for STAT1 in G-CSF-driven myeloid cell proliferation is unlikely, given the normal production of granulocytes in STAT1-deficient mice [20]. STAT3 and SOCS3 (viz. a suppressor of cytokine signalling, the expression of which is induced by STAT3) are negative regulators of granulopoiesis. Indeed, mice lacking any of these proteins in their hematopoietic compartment develop neutrophilia, and their bone marrow cells are hyperresponsive to G-CSF stimulation [21], [22]. Several lines of evidence suggest that STAT5 is implicated in neutrophil development. First, loss of STAT5 function in primary bone marrow cells leads to a reduction in CFU-G colony formation in vitro [23], [24]. Second, STAT5 favors the survival of myeloid progenitors by inducing the expression of Bcl-x_L_, an antiapoptotic protein of the Bcl-2 family [25]. Third, bone marrow cells from mice lacking both STAT5 isoforms, STAT5a and STAT5b, are unable to repopulate the neutrophilic granulocyte lineage of lethally irradiated wild-type hosts [26], [27]. Disconcertingly, however, STAT5ab^−/−^ mice spontaneously develop mild neutrophilia [23], [27].

The purpose of the current study was to definitively clarify the role of STAT5 in neutrophil homeostasis. To determine the net effects of STAT5 proteins on neutrophil homeostasis, we have chosen to use the STAT5a/b double-knockout mice (23; hereafter referred to as STAT5^−/−^ mice).

## Results

### Increased Numbers of Functional Neutrophils in the Periphery of STAT5^−/−^ Mice

We first examined the peripheral blood counts of wild-type and STAT5^−/−^ mice. The total leukocyte counts in STAT5^−/−^ animals were about 2-fold lower than in wild-type controls. This effect was largely due to lymphopenia. However, STAT5^−/−^ animals displayed mild neutrophilia (3.2±1.0×10^3^ neutrophils/µl in STAT5^−/−^ mice versus 1.6±0.4×10^3^ neutrophils/µl in wild-type controls, n = 22, *P*<.001) as well as mild monocytosis, a finding that is in accordance with previous reports [23], [27].

**Figure 1:**
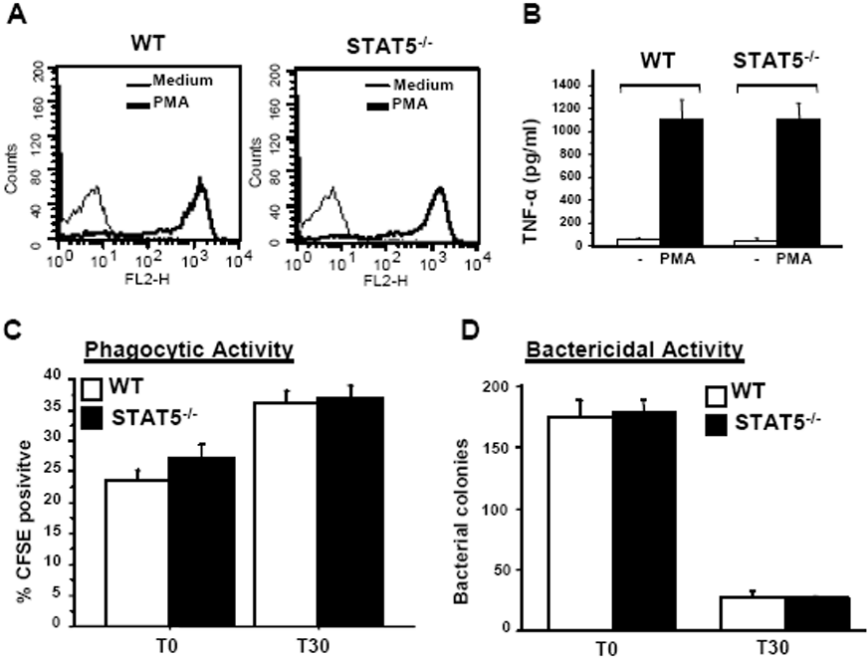
Peripheral STAT5^−/−^ neutrophils are functional. (A) Peritoneal neutrophils from TGA-treated wild-type (WT) or STAT5^−/−^ mice were analyzed for oxidation of dihydrorhodamine to fluorescent rhodamine by FACS with or without treatment with PMA (1 µg/ml). (B) STAT5^−/−^ neutrophils are able to produce TNF-α. 1×10^6^ peritoneal neutrophils from control or mutant mice were stimulated for 6 h with PMA (1 µg/ml), and TNF-α concentration in cell supernatants was then measured by ELISA. (C) STAT5^−/−^ neutrophils are phagocytic. CFSE-labeled *Staphylococcus aureus* were incubated with peritoneal neutrophils at 37°C for 30 min (T0), and gentamycin was added for an additional 30 min (T30). Cells washed free of extracellular bacteria at T0 or T30 min were analyzed by FACS for engulfed bacteria. (D) STAT5^−/−^ neutrophils are bactericidal. Engulfed bacteria were released by lysing peritoneal neutrophils in 1 ml water. One hundred microliters of a 1:1,000 dilution of the bacterial suspension was plated and colonies were counted after a 24-h incubation at 37°C.

Neutrophil migration in STAT5^−/−^ mice was examined in a thioglycollate (TGA)-induced peritonitis model. At 4 h after TGA injection, the absolute number of neutrophils in the peritoneal cavity of STAT5^−/−^ animals was about 2-fold higher than in wild-type littermates. Moreover, neutrophils isolated from the peritoneal space of TGA-treated STAT5^−/−^ mice were morphologically mature and stained normally for Gr-1 and CD11b, two late-stage markers of neutrophil differentiation (data not shown). Finally, STAT5^−/−^ peritoneal neutrophils displayed a respiratory oxidative burst, and were able to produce high quantities of TNF-α and to phagocytose and kill bacteria (Figures 1A–D).

Taken together, these results confirm that STAT5^−/−^ mice spontaneously develop mild neutrophilia and show that STAT5^−/−^ neutrophils are functional.

### Neutrophilia in STAT5^−/−^ mice is due to increased granulopoiesis

**Figure 2:**
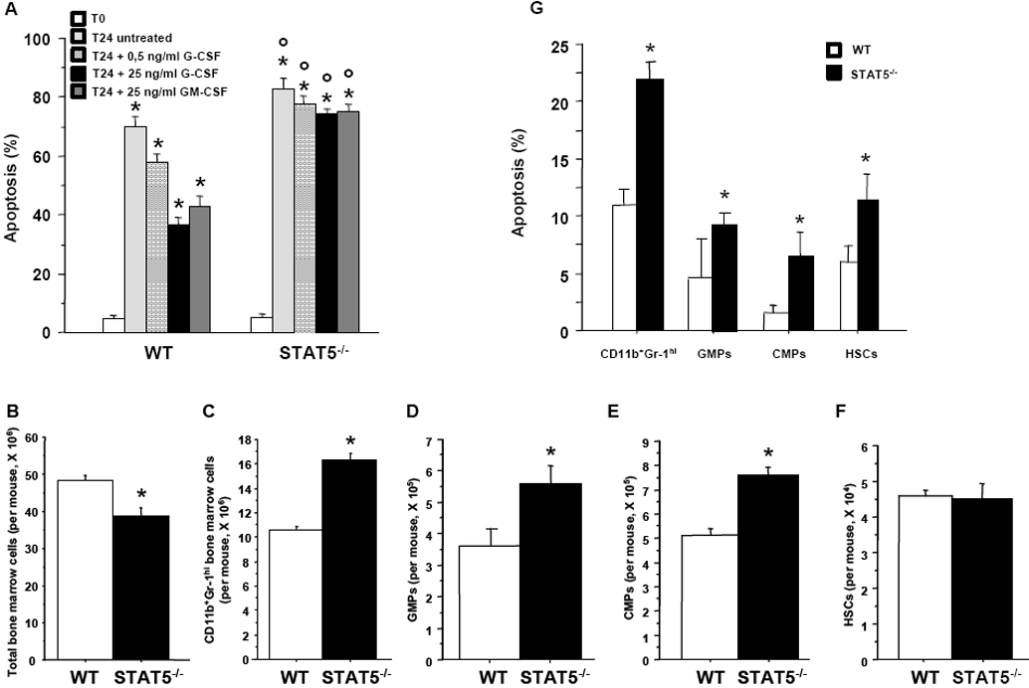
Increased granulopoiesis in STAT5^−/−^ mice. (A) Apoptosis of peripheral neutrophils. MACS-purified blood peripheral neutrophils from wild-type (WT) and STAT5^−/−^ mice were cultured for 24 hr in the absence or presence of 0.5 or 25 ng/ml G-CSF or 25 ng/ml GM-CSF. Freshly purified (T0) and 24-hr cultured (T24) neutrophils were assayed for apoptosis using dual color annexin-V-FITC/propidium iodide staining and flow cytometry analyses. *, significantly different from T0 values with P<0.05. °, significantly different from WT values with P<0.05. (B) Bone marrow was harvested from both hind limbs of either WT or STAT5^−/−^ mice, and cell counts were determined after red blood cell lysis. (C–F) Mature marrow neutrophils (CD11c^+^Gr-1^hi^ cells), GMPs (Lin^−^Sca-1^−^IL-7Rα^−^c-kit^+^CD34^+^FcγR^+^ cells), CMPs (Sca-1^−^IL-7Rα^−^c-kit^+^CD34^+^FcγR^low^ cells), and HSCs (Lin^−^Sca-1^hi^IL-7Rα^−^c-kit^hi^) from the bone marrow of control and mutant mice were counted by flow cytometry. Absolute values were generated by multiplying gated percentages by total cell numbers. (G) Freshly FACS-purified marrow neutrophils, GMPs, CMPs, and HSCs were assayed for apoptosis using trypan blue exclusion. (B–G) *, significantly different from WT values with P<0.05.

We hypothesized that neutrophilia in STAT5^−/−^ mice could be due to enhanced survival of peripheral neutrophils, to increased granulopoiesis, or to a combination of these two mechanisms. Freshly purified blood neutrophils from STAT5^−/−^ and control mice displayed low and similar levels of apoptosis (Figure 2A). After 24 h of culture, apoptosis levels were substantially increased and were significantly greater in neutrophils from STAT5^−/−^ mice than in those from wild-type controls (Figure 2A), invalidating the hypothesis that neutrophilia in STAT5^−/−^ mice is the consequence of enhanced survival of peripheral neutrophils. Blood neutrophils were also cultured for 24 h in the presence of G-CSF or GM-CSF, two antiapoptotic cytokines [16]. Both G-CSF and GM-CSF were able to partially inhibit apoptosis of wild-type neutrophils, whereas STAT5^−/−^ neutrophils did not respond to these cytokines (Figure 2A).

We next examined whether neutrophil production was increased in STAT5^−/−^ mice. Although STAT5^−/−^ mice showed a ∼20% reduction in the total number of bone marrow cells (Figure 2B), the absolute number of mature neutrophils (CD11b^+^Gr-1^hi^) in the bone marrow of these mice was significantly increased relative to wild-type littermates (Figure 2C). The CMP, the GMP, the myeloblast, the promyelocyte, the myelocyte, and the metamyelocyte populations were also substantially increased, whereas the HSC population was unchanged, in STAT5^−/−^ animals relative to wild-type controls (Figures 2D–2F; data not shown). Finally, we found that the apoptosis rate of FACS-purified HSCs, CMPs, GMPs, and marrow neutrophils was higher in STAT5^−/−^ mice than in controls (Figure 2G). Altogether, these results indicate that neutrophilia in STAT5^−/−^ mice is due to increased granulopoiesis rather than to enhanced neutrophil survival.

### Increased granulopoiesis in STAT5^−/−^ mice is associated with elevated G-CSF production

**Figure 3:**
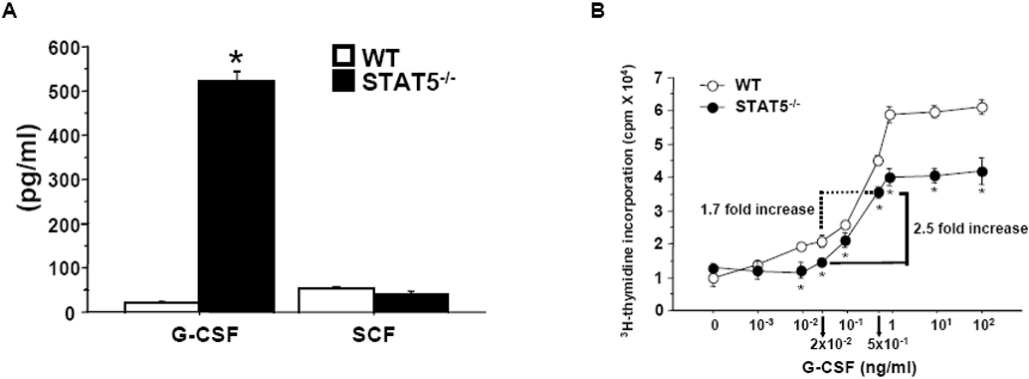
Association between increased granulopoiesis and elevated G-CSF production in STAT5^−/−^ mice. (A) Elevated G-CSF serum levels in STAT5^−/−^ mice. G-CSF and SCF concentrations in the serum of wild-type (WT) and STAT5^−/−^ mice were determined by ELISA. *, significantly different from WT values with P<0.05. (B) Proliferation of bone marrow cells from wild-type and mutant mice in response to G-CSF. 2×10^5^ STAT5^−/−^ or control bone marrow cells were cultured with G-CSF in various concentrations for 48 hr before cells were pulsed with ^3^[H]thymidine for 16 hr. Note that mutant cells stimulated with 5×10^−1^ ng/ml G-CSF displayed a 1.7-fold and a 2.5-fold increase in proliferation, respectively, compared with control cells and mutant cells stimulated with 2×10^−2^ ng/ml G-CSF. (A, B) *, significantly different from WT values with P<0.05.

To investigate whether increased neutrophil production in STAT5^−/−^ mice could be the consequence of an aberrant production of cytokines involved in granulopoiesis, we measured the concentrations of G-CSF, GM-CSF, IL-3, and stem cell factor (SCF) in the serum of wild-type and mutant mice. Both wild-type and STAT5^−/−^ mice had undetectable serum GM-CSF and IL-3. Serum levels of SCF were similar in both mice, whereas G-CSF levels were ∼25-fold higher in STAT5^−/−^ mice than in controls (Figure 3A). Such a phenomenon could explain why STAT5^−/− ^mice display enhanced granulopoiesis and develop neutrophilia. To test this hypothesis, bone marrow cells were isolated from wild-type and mutant mice, and their proliferative response to G-CSF was assayed. As illustrated in Figure 3B, the proliferative response of STAT5^−/−^ cells to G-CSF was significantly reduced relative to that of control cells. However, STAT5^−/−^ bone marrow cells stimulated in vitro with G-CSF at a concentration equivalent to that measured in the serum of STAT5^−/−^ mice (500 pg/ml) displayed a 1.7-fold and a 2.5-fold increase in proliferation, respectively, compared with wild-type cells and mutant cells stimulated with G-CSF at a physiological concentration (20 pg/ml). Thus, although the proliferative response of mutant bone marrow cells to G-CSF is reduced, the increased neutrophil production observed in STAT5^−/−^ mice is recapitulated ex vivo by stimulating the cells with appropriate G-CSF concentrations. In this experiment, we have hypothesized that the concentration of G-CSF in the bone marrow was equivalent to that in the serum.

Taken together, these data show that G-CSF production is increased in STAT5^−/−^ mice, allowing mutant progenitors to overcompensate for their hyporesponsiveness to this cytokine.

### STAT5^−/−^ liver endothelial cells autonomously produce high quantities of G-CSF

**Figure 4:**
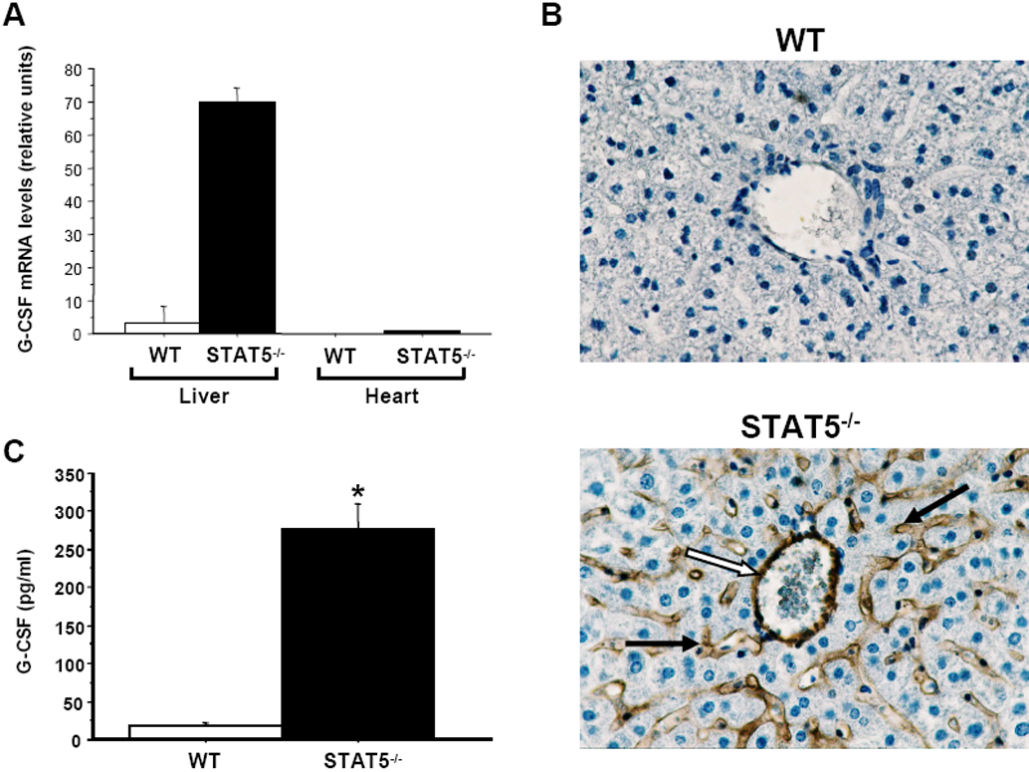
STAT5^−/−^ LECs produce high amounts of G-CSF. (A) Elevated G-CSF mRNA expression in the liver of STAT5^−/−^ mice. Liver and heart from wild-type (WT) and STAT5^−/−^ mice were assayed for G-CSF mRNA expression by real time quantitative RT-PCR. All values are normalized to β–actin mRNA. No G-CSF mRNA was detected in WT heart. (B) LECs are the source of G-CSF in STAT5^−/−^ mice. Liver sections from WT and mutant mice were stained to detect G-CSF (brown). Both endothelial cells lining the central vein (open arrow) and sinusoidal endothelial cells (solid arrow) stained for G-CSF. Original magnification, 250×. (C) STAT5^−/−^ LSECs autonomously produce high quantities of G-CSF. WT and STAT5^−/−^LSECs were isolated, seeded onto 6-well flat-bottom plates at a density of 5×10^6^ cells/well, and cultured for 3 days after which the medium was refreshed. Supernatants were harvested 6 hr later and analyzed for G-CSF concentration by ELISA. *, significantly different from WT values with P<0.05.

To identify the source of G-CSF in vivo, we first analyzed various organs and tissues from wild-type and STAT5^−/−^ mice for their relative expression of G-CSF mRNA. In both control and mutant mice, the G-CSF transcript was predominantly detected in the liver (Figure 4A). However, G-CSF mRNA levels were ∼15-fold higher in STAT5^−/−^ than in normal liver. G-CSF mRNA was also detected at low levels in the heart of mutant mice. No G-CSF mRNA was found in intestines, lungs, kidneys, skin, spleen, bone marrow, lymph nodes, and white blood cells from control and STAT5^−/−^ mice. Immunohistochemistry revealed that G-CSF protein expression was restricted to endothelial cells lining the central veins and sinusoidal endothelial cells (both these cell types are hereafter referred to as liver endothelial cells, LECs) in the liver of STAT5^−/−^ mice (Figure 4B, lower panel). In wild-type mice, liver cells did not stain for G-CSF (Figure 4B, upper panel), except in some fields where G-CSF-positive LECs could be detected (data not shown). Of note, some endothelial cells staining for G-CSF were found in auricles and aorta of STAT5^−/−^ mice (data not shown). No signal was found in any of the other organs (data not shown).

We next sought to determine whether LECs from STAT5^−/−^ mice were able to autonomously secrete G-CSF. Control and STAT5^−/−^ liver sinusoidal endothelial cells (LSECs) were isolated and cultured for 3 days. The medium was then replaced by fresh medium and the cells were cultured for an additional 6 h, after which supernatants were harvested and analyzed for G-CSF concentration by ELISA. G-CSF was barely detectable in the supernatants of wild-type LSECs (Figure 4C). Conversely, significant levels of G-CSF were found in the supernatants of mutant cells, indicating that STAT5^−/−^ LSECs are capable of releasing high amounts of G-CSF without external stimulation.

### STAT5b is rapidly degraded in wild-type LECs following inflammatory stimulation

**Figure 5:**
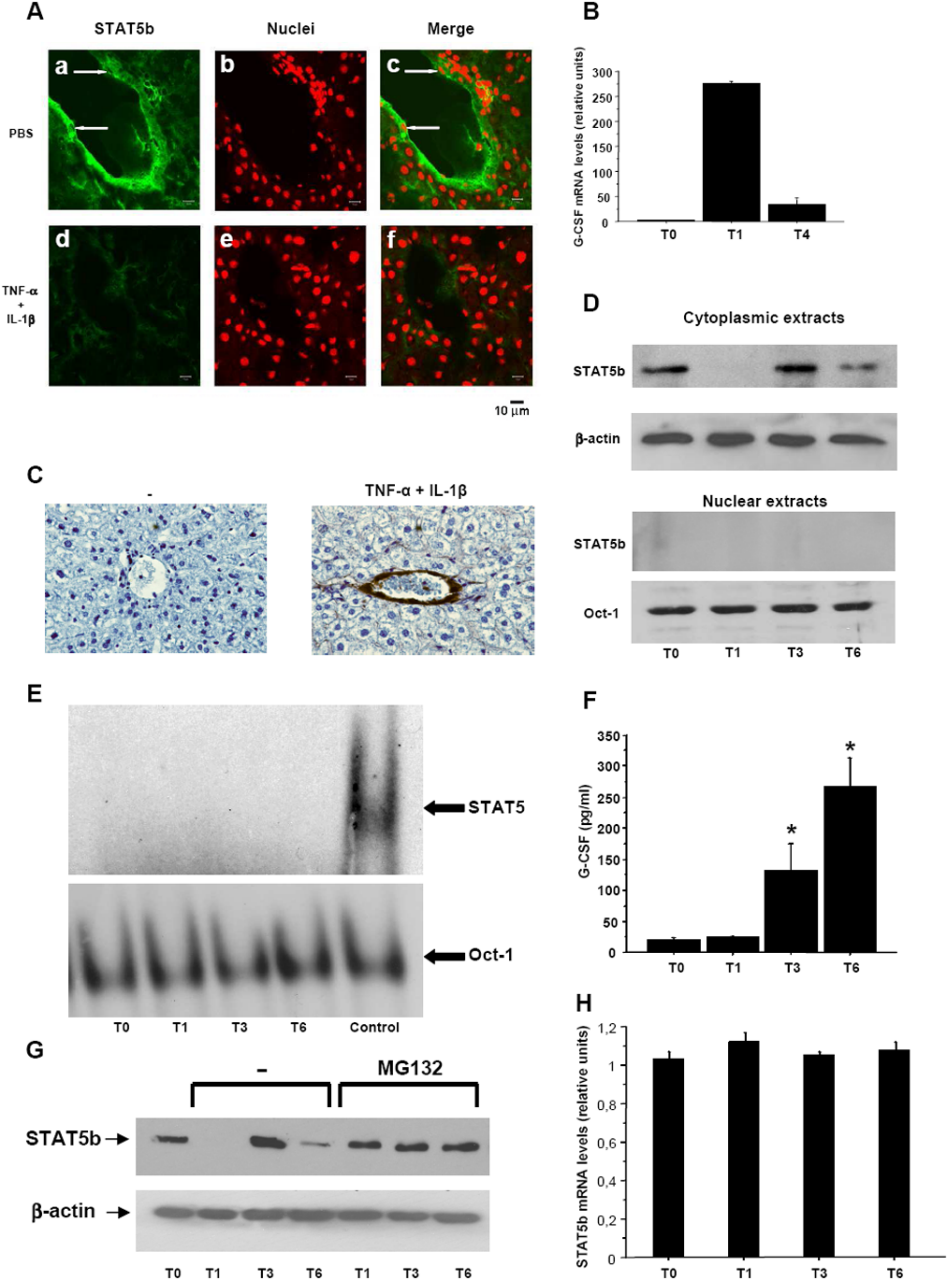
Degradation of cytoplasmic STAT5b in WT LECs following inflammatory stimulation, and subsequent enhancement of G-CSF production. (A) Wild-type mice were injected intravenously with PBS or with 2 µg recombinant murine TNF-α and 2 µg recombinant murine IL-1β. Livers were harvested 30 min later, and liver sections were analyzed for STAT5b expression and localization by immunofluorescence and confocal microscopy as described in the Experimental Procedures. (a) STAT5b was present in the cytoplasm (green), but not the nucleus (arrows), of LECs of PBS-treated mice. (d) STAT5b was no longer detectable in LECs of cytokine-treated mice. All cell nuclei in the fields are shown by hexidium iodide staining (red, b and e), and merges of frames (a) and (b), and (d) and (e), are given in (c) and (f), respectively. Bars = 10 µm. (B) Livers collected from cytokine-treated mice were assayed for G-CSF mRNA expression by quantitative RT-PCR. Livers were harvested before cytokine injection (T0), or either 1 (T1) or 4 (T4) hr postinjection. All values are normalized to β-actin mRNA. (C) Liver sections from untreated mice (left panel) and mice treated for 4 hr with TNF-α and IL-1β (right panel) were stained to visualize G-CSF (brown). Original magnification, 250×. (D) LSECs were isolated from wild-type mice and cultured for 3 days before experiments were performed. Cells were treated for 0 (T0), 1 (T1), 3 (T3) or 6 (T6) hr with 100 IU/ml TNF-α and 500 pg/ml IL-1β. Cytoplasmic and nuclear extracts were prepared and analyzed for STAT5b expression by immunoblot. Equal loading of proteins on the gel was confirmed by probing the blots for β-actin (cytoplasmic extracts) or Oct-1 (nuclear extracts). (E) Nuclear protein extracts were assessed for STAT5 DNA-binding activity by EMSAs. Binding of Oct-1 was used as an internal standard. Nuclear extracts from prolactin-stimulated bovine MAC-T cells were used as positive controls for STAT5 activation (control). (F) G-CSF concentrations in LSEC supernatants were measured by ELISAs. *, significantly different from T0 values with P<0.05. (G) LSECs were isolated from wild-type mice and cultured for 3 days before experiments were conducted. Cells were treated with 20 µM MG132 for 30 min prior to stimulation with 100 IU/ml TNF-α and 500 pg/ml IL-1β. Cytoplasmic extracts were prepared 0 (T0), 1 (T1), 3 (T3), and 6 (T6) hr later and analyzed for STAT5b expression by immunoblot. Equal loading of proteins on the gel was confirmed by probing the blots for β-actin. (H) LSECs stimulated for 0 (T0), 1 (T1), 3 (T3), and 6 (T6) hr with 100 IU/ml TNF-α and 500 pg/ml IL-1β were assayed for STAT5b mRNA expression by real-time quantitative RT-PCR. All values were normalized to β-actin mRNA.

To assess whether our observations in STAT5^−/−^ mice are of physiological relevance, we investigated the expression and activation of STAT5 in wild-type LECs under steady-state conditions and following inflammatory stimulation. First, confocal microscopy was used to visualize STAT5a and STAT5b in liver sections from wild-type mice. Under physiological conditions, STAT5b was expressed at high levels in LECs, but was barely detectable in other liver cells (Figure 5Aa). STAT5a was not present in LECs (data not shown). Of note, STAT5b was exclusively located in the cytoplasm of LECs; no staining was ever observed in the nucleus (Figure 5Aa, arrows). Thirty minutes after intravenous injection of wild-type mice with TNF-α and IL-1β, STAT5b was no longer detected in LECs (Figure 5Ad), indicating that inflammatory stimulation induces rapid degradation of cytoplasmic STAT5b in these cells. Subsequent to STAT5b degradation, G-CSF mRNA and protein expression significantly increased in the liver, as determined by real time quantitative RT-PCR and immunohistochemistry (Figures 5B and C). G-CSF staining was also observed in heart endothelium and liver, lung, kidney, and spleen macrophages following inflammatory stimulation (data not shown).

To examine direct effects of inflammatory stimulation on STAT5 expression, LSECs were isolated from wild-type mice and activated ex vivo with TNF-α and IL-1β. Western blotting revealed the presence of STAT5b, but not STAT5a, in cytoplasmic extracts from wild-type LSECs at steady-state (Figure 5D). 1 h after TNF-α and IL-1β stimulation, STAT5b was no longer detectable in the cytoplasm of LSECs (Figure 5D). As STAT5b was not present in nuclear extracts at any time point (Figure 5D), we concluded that STAT5b was degraded rather than translocated to the nucleus. After 3 h, STAT5b was resynthesized, but after 6 h of activation, STAT5b was found to be degraded again. Absence of STAT5b nuclear translocation and DNA-binding activity was confirmed by EMSAs (Figure 5E), and the ability of the anti-STAT5b antibody to recognize phosphorylated STAT5b was checked by performing immunoblots with nuclear extracts from GM-CSF-treated murine neutrophils (data not shown), in which STAT5b is known to be rapidly phosphorylated and translocated to the nucleus [28]. Finally, ELISAs showed that G-CSF production by LSECs was increased at 3 and at 6 h poststimulation (Figure 5F). STAT5b was already degraded at 1 h poststimulation (Figure 5D). However, G-CSF production was only detected after 3 h, when STAT5b was resynthetized (Figures 5D and 5F). This apparent discrepancy may be explained by the fact that there is a delay i) between STAT5b degradation and G-CSF release (this delay corresponds to the time required for G-CSF transcription, translation and secretion) and ii) between STAT5b resynthesis and complete inhibition of G-CSF expression (this delay correspond to the time needed for the elimination of residual G-CSF transcripts). It has been reported that cytoplasmic STAT5 may be ubiquitinated and degraded by the proteasome [29]. To determine whether inflammatory stimulation leads to proteasomal degradation of cytoplasmic STAT5b in liver endothelium, LSECs were isolated and treated with the proteasome inhibitor MG132 30 min prior to stimulation with TNF-α and IL-1β. Cytoplasmic extracts were prepared 1, 3, and 6 hr later and analyzed for STAT5b expression by immunoblot. Figure 5G shows that MG132 completely prevented the disappearance of cytoplasmic STAT5b in stimulated LSECs, unambiguously demonstrating that in these cells, cytoplasmic STAT5b is degraded by the proteasome. Moreover, real-time quantitative RT-PCR revealed that STAT5b mRNA levels were not altered in TNF-α- and IL-1β-stimulated LSECs (Figure 5H), indicating that transcriptional or post-transcriptional mechanisms do not account for the decrease in cytoplasmic STAT5b protein levels observed in LSECs upon inflammatory stimulation. Altogether, these data demonstrate that STAT5b is rapidly degraded by the proteasome in LECs following inflammatory stimulation, and that STAT5b degradation precedes enhancement of G-CSF production by these cells.

### Neutrophil homeostasis cannot be maintained in STAT5^−/−^ mice upon inflammatory stimulation

**Figure 6:**
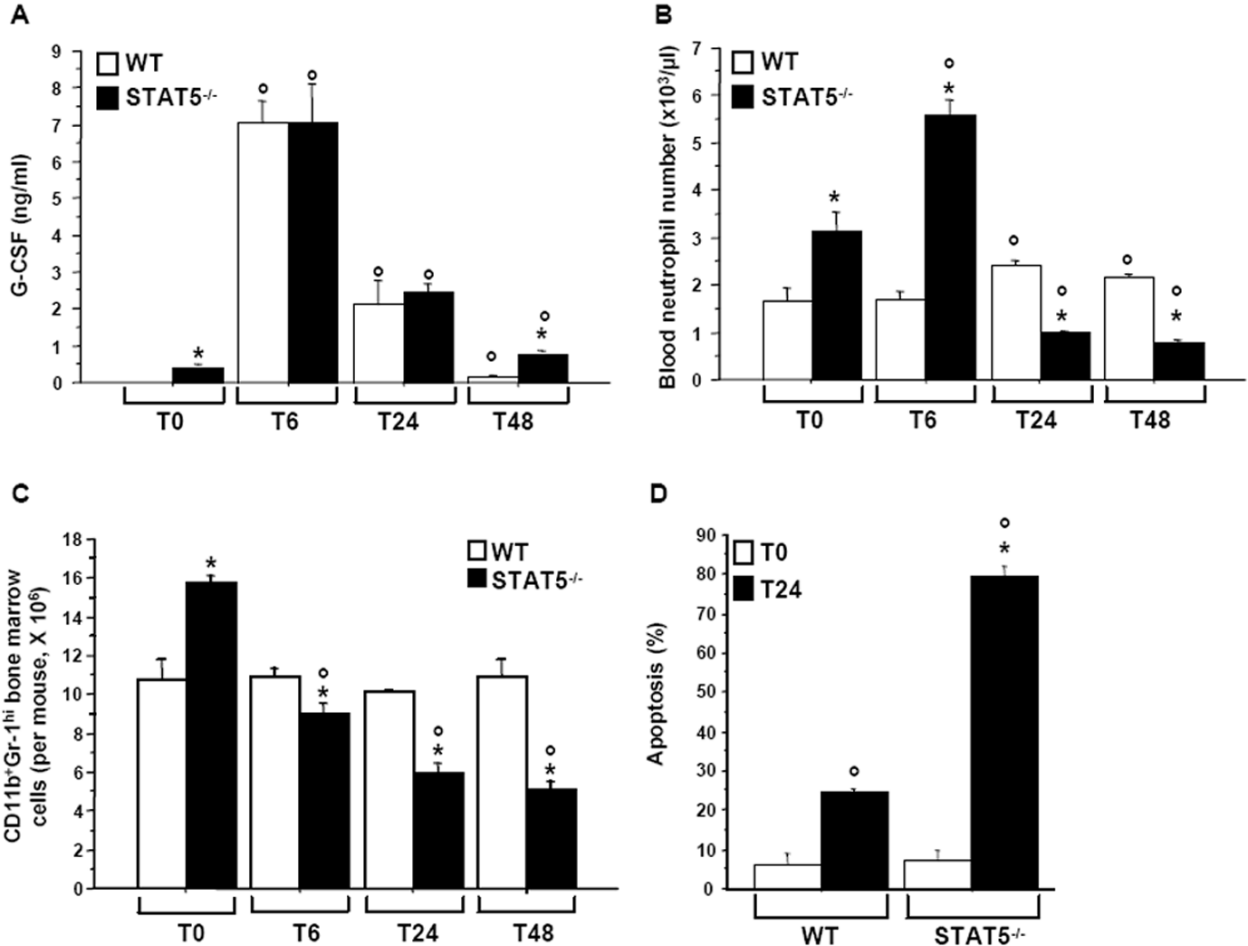
STAT5^−/−^ mice are unable to maintain neutrophil homeostasis during cytokine-induced inflammation. (A) Wild-type (WT) and mutant mice were injected intravenously with 2 µg recombinant murine TNF-α and 2 µg recombinant murine IL-1β. Serum G-CSF concentrations were measured by ELISA before (T0) and 6 (T6), 24 (T24), and 48 (T48) hr after cytokine injection. (B) Blood neutrophils were counted at the different time points. (C) Mature marrow neutrophils (CD11c^+^Gr-1^hi^ cells) from control and mutant mice were counted by flow cytometry. Absolute values were generated by multiplying gated percentages by total cell numbers. (D) Blood neutrophils were isolated by MACS from wild-type and mutant mice 24 hr after cytokine injection. Freshly purified (T0) and 24-hr cultured (T24) neutrophils were assayed for apoptosis using dual color annexin-V-FITC/propidium iodide staining and flow cytometry analyses. (A–D) *, significantly different from WT values with P<0.05. °, significantly different from T0 values with P<0.05.

We next compared the inflammatory response in STAT5^−/−^ mice with that in wild-type mice. Inflammation was provoked by intravenous injection of IL-1β and TNF-α. Serum G-CSF concentrations increased substantially in both wild-type and mutant mice during the first 6 h after injection, and then gradually decreased to reach basal values by 48 h (Figure 6A). In wild-type mice, blood neutrophil numbers were slightly, but significantly, increased at 24 and 48 h after cytokine administration (Figure 6B). In STAT5^−/−^ mice, although a ∼2-fold rise in blood neutrophil counts was observed at 6 h, neutrophil numbers dropped to only 31 and 25% of the initial values at 24 and 48 h postinjection, respectively (Figure 6B).

During the inflammatory response, both neutrophil production and survival are enhanced [1], [5], [6], allowing continuous replacement of extravasated neutrophils. We thus investigated whether these homeostatic mechanisms were deficient in STAT5^−/−^ mice during cytokine-induced inflammation. The absolute number of bone marrow neutrophils remained constant throughout the experimental period in wild-type mice, whereas marrow neutrophil counts in mutant mice gradually declined to less than one third of basal values at 48 h (Figure 6C). Moreover, blood neutrophils recovered from wild type mice 24 h after cytokine injection displayed enhanced survival, while neutrophils from STAT5^−/−^ mice did not (Figure 6D; compare with Figure 2A). Therefore, when injected with pro-inflammatory cytokines, mutant mice cannot increase neutrophil production and survival, and are therefore unable to maintain neutrophil homeostasis.

### Stromal cell-specific loss of STAT5 induces marked neutrophilia

**Figure 7:**
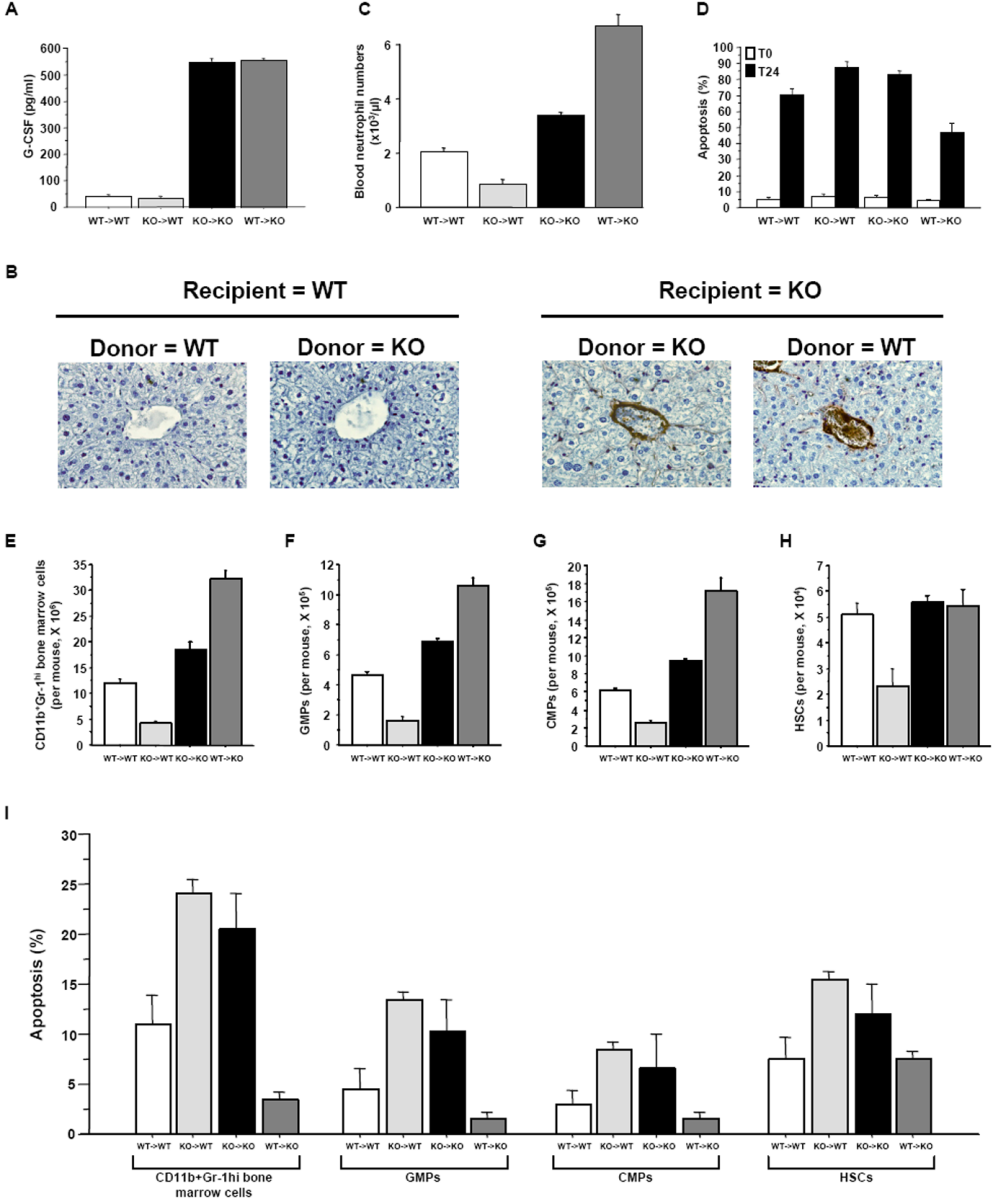
Stromal cell-specific loss of STAT5 induces prominent neutrophilia. (A) 5×10^6^ wild-type bone marrow cells were injected intravenously into lethally irradiated (1,000 rad) STAT5^−/−^ mice (WT→KO) and vice versa (KO→WT). 8 weeks later, serum G-CSF concentrations were measured by ELISA. Lethally irradiated wild-type mice reconstituted with wild-type bone marrow (WT→WT) and mutant mice reconstituted with mutant marrow (KO→KO) served as controls. (B) Liver sections were stained to detect G-CSF (brown). Original magnification, 250×. (C) Blood neutrophils were counted. (D) Freshly MACS-purified (T0) and 24-hr cultured (T24) blood neutrophils were assayed for apoptosis using dual color annexin-V-FITC/propidium iodide staining and flow cytometry analyses. (E–H) Mature marrow neutrophils, GMPs, CMPs, and HSCs were counted by flow cytometry. Absolute values were generated by multiplying gated percentages by total cell numbers. (I) Freshly FACS-purified marrow neutrophils, GMPs, CMPs, and HSCs were assayed for apoptosis using trypan blue exclusion.

Based on the data presented above, we postulated that extinction of STAT5 expression in LECs but not in cells of the granulocytic lineage would induce prominent neutrophilia. To test the validity of our hypothesis, we generated chimeric mice by adoptive bone marrow transplantation after total body irradiation. Bone marrow cells from control wild-type mice were transferred into lethally irradiated STAT5^−/−^ recipients (WT→KO) and vice versa (KO→WT). WT→WT and KO→KO mice were used as controls. Both KO→KO and WT→KO mice displayed elevated G-CSF serum levels, most likely due to the fact that their LECs were capable of producing large amounts of this cytokine (Figures 7A and 7B). The phenotype of WT→WT and KO→KO mice recapitulated that of primary wild-type and mutant mice, respectively (Figures 7C–I; compare with Figure 2). In KO→WT mice, total numbers of HSCs, CMPs, GMPs, and bone marrow and peripheral blood neutrophils were substantially reduced relative to those in WT→WT and KO→KO mice (Figures 7C and 7E–H). Interestingly, however, apoptosis of HSCs, CMPs, GMPs, and marrow and blood neutrophils was only slightly increased in KO→WT mice as compared with KO→KO counterparts in which G-CSF concentrations are much higher (Figures 7D and 7I). As expected, WT→KO mice displayed a significant increase in blood neutrophil counts when compared with WT→WT mice (Figure 7C). Of note, the WT→KO mice had blood lymphocytes in numbers similar to those in WT→WT mice (data not shown). The pronounced neutrophilia found in WT→KO mice was associated with substantial enhancement of blood neutrophil survival (Figures 7D). Moreover, a significant rise in total numbers and survival of bone marrow neutrophils, GMPs, CMPs and HSCs was noted in KO→WT mice compared to WT→WT controls (Figures 7E–I). These results demonstrate that loss of STAT5 expression in stromal cells, and especially in LECs, is sufficient to induced marked neutrophilia.

## Discussion

Differentiation of blood cells from HSCs is controlled by lineage-restricted transcription factors [30]. Several lines of evidence suggest that STAT5 is an essential component of G-CSF-driven cell proliferation and granulopoiesis [23], [24], [26], [27]. Surprisingly, however, STAT5^−/−^ mice develop mild neutrophilia rather that neutropenia [23], [27]. In the present study, we provide an explanation for these conflicting results. Indeed, we find that in STAT5^−/−^ mice, LECs autonomously produce high amounts of G-CSF, allowing bone marrow progenitors to overcompensate for their hyporesponsiveness to this cytokine.

### STAT5 Functions in the Granulocytic Lineage

Apoptosis of HSCs, CMPs, GMPs, and bone marrow neutrophils was increased in STAT5^−/−^ mice, an observation consistent with previous reports that survival of lin^dim^Sca-1^+^ and lin^dim^Sca-1^neg/lo^ bone marrow cells is decreased in the absence of STAT5 [26], and that STAT5 may promote the survival of myeloid progenitors through its ability to induce transcription of the *bcl-x* gene [25]. However, the HSC population was unchanged, and the CMP, GMP, and bone marrow neutrophil populations were increased in mutant mice compared with wild-type controls. To determine the contribution of elevated G-CSF production by STAT5^−/−^ LECs to this phenotype, we have generated KO→WT mice. In KO→WT mice, HSC, CMP, GMP, and bone marrow neutrophil apoptosis was only slightly augmented relative to that in KO→KO control mice, whereas total HSC, CMP, GMP, and bone marrow neutrophil counts were dramatically reduced. These results are consistent with a model in which increased G-CSF production by LECs in STAT5^−/−^ mice triggers STAT5-independent compensatory mechanisms in various progenitors. These mechanisms mask the intrinsic defect in STAT5^−/−^ progenitor survival and direct the commitment of progenitors to the granulocytic lineage. The main action of G-CSF is not through induction of competing antiapoptotic programs. Indeed, only moderate increase in progenitor apoptosis was observed in the absence of elevated G-CSF concentrations (viz. in KO→WT mice). It is thus probable that G-CSF exerts its effects by enhancing progenitor proliferation. This hypothesis is supported by two independent observations. First, both the lin^dim^Sca-1^+^ and the lin^dim^Sca-1^neg/lo^ subsets exhibit a 2-fold increase in the percentage of cells with DNA content more than 2n in STAT5^−/−^ mice [26]. Second, STAT5^−/−^ bone marrow cells stimulated in vitro with G-CSF at a concentration equivalent to that measured in the serum of STAT5^−/−^ mice (500 pg/ml) displayed a 1.7-fold and a 2.5-fold increase in proliferation, respectively, compared with wild-type cells and mutant cells stimulated with G-CSF at a physiological concentration (20 pg/ml) (Figure 3B). Further experiments are required to determine whether G-CSF is the sole factor responsible for the neutrophilia in STAT5^−/−^ mice.

Neutrophil homeostasis was not maintained in STAT5^−/−^ mice following intravenous injection of TNF-α and IL-1β. Indeed, although a rise in blood neutrophil counts was observed at 6h postinjection, neutrophil numbers dropped significantly after 24 and 48 h. Maintenance of the neutrophil pool size during the inflammatory response requires enhanced neutrophil production [6]. G-CSF is the primary regulator of emergency granulopoiesis [9]–[11]. Serum G-CSF levels increased from ∼500 to 7,000 pg/ml in STAT5^−/−^ mice injected with pro-inflammatory cytokines. However, the total number of bone marrow CD11b^+^Gr-1^hi^ cells declined concurrently to less than one third of basal values, indicating that neutrophil release from bone marrow cannot be compensated by efficient emergency granulopoiesis in these mice. It is possible that the survival defect in STAT5^−/−^ bone marrow progenitors prevented the enhancement of neutrophil production that is normally induced by increasing G-CSF concentrations. This hypothesis is supported by our finding that proliferation of STAT5^−/−^ bone marrow cells plateaued at approximately 500 pg/ml G-CSF, which is the concentration measured in the serum of STAT5^−/−^ mice at steady state (Figure 3B). In wild-type mice, inflammatory stimulation induced G-CSF production by LECs and other cell types (principally macrophages), and thus sustained neutrophil mobilization and expansion. We conclude that STAT5-mediated antiapoptosis in myeloid progenitors is required for efficient emergency granulopoiesis.

Blood neutrophils from STAT5^−/−^ mice were fully differentiated. Moreover, STAT5^−/−^ neutrophils displayed a respiratory oxidative burst, increased expression of surface markers following stimulation, and the ability to migrate, to produce high quantities of TNF-α, and to phagocytose and kill bacteria. STAT5 is therefore dispensable for neutrophil differentiation and function.

Of all the cells of the immune system, the neutrophil has the shortest half-life, estimated to be between 6 and 18 h. This short half-life arises from the fact that bloodstream neutrophils rapidly undergo spontaneous apoptosis [1]. The apoptosis rate was slightly but significantly greater in peripheral blood neutrophils from STAT5^−/−^ mice than in those from wild-type littermates, indicating that at steady state, STAT5 contributes to homeostatic regulation of peripheral blood neutrophil numbers by inducing antiapoptotic mechanisms not only in progenitors but also in circulating neutrophils.

Delayed neutrophil apoptosis has been associated with several infectious and inflammatory diseases [31]. This enhancement of neutrophil survival presumably allows them to function more effectively at sites of inflammation. The suppression of neutrophil apoptosis in vivo is mainly due to overexpression of survival factors, two most important of which are G-CSF and GM-CSF [5], [32]. In our study, STAT5^−/−^ neutrophils, unlike wild-type neutrophils, were refractory to G-CSF and GM-CSF-induced antiapoptosis in vitro. Moreover, survival of circulating neutrophils was not enhanced in STAT5^−/−^ mice following inflammatory stimulation, indicating that STAT5 is a critical mediator of delayed neutrophil apoptosis in vivo. In chronic inflammatory diseases, accumulation of long-lived neutrophils at the site of inflammation is deleterious rather than beneficial. We postulate that selective inhibition of STAT5 could have therapeutic value in the control of these disorders.

### Functions of STAT5b in LSECs

The most striking finding of our work is that LECs and, to a lesser extent, auricle and aorta endothelial cells of STAT5^−/−^ mice autonomously secrete G-CSF. This situation is recapitulated in wild-type mice upon inflammatory stimulation. Indeed, rapid degradation of STAT5b, which is the sole STAT5 isoform present in LECs, precedes G-CSF expression and secretion by LECs in wild-type mice injected with TNF-α and IL-1β. To examine direct effects of inflammatory stimulation on STAT5b protein expression, isolated wild-type LSECs were treated ex vivo with TNF-α and IL-1β. Again, STAT5b was rapidly degraded following pro-inflammatory cytokine stimulation. Taken together, these data show that STAT5b negatively regulates granulopoiesis at steady-state by directly or indirectly repressing G-CSF expression in liver endothelium, and that cytokine-induced removal of this STAT5b-imposed brake triggers G-CSF production and secretion. To address the question of whether enhancement of G-CSF production that results from STAT5b degradation in LECs may contribute to induction of emergency granulopoiesis, we have generated WT→KO mice. In these mice, bone marrow-derived cells were normal, whereas LECs were deficient for STAT5 and thus produced large amounts of G-CSF. Total numbers and survival of CMPs, GMPs, and bone marrow and peripheral blood neutrophils were significantly increased in WT→KO mice relative to WT→WT control mice, demonstrating that loss of STAT5 expression in LECs is sufficient to provoke emergency granulopoiesis and eventually neutrophilia. We conclude that in LECs, STAT5b is a stress sensor, the degradation of which allows conversion of systemic inflammatory signals into signals that promote granulopoiesis. Of note, although chimeric WT→KO mice had normal numbers of blood lymphocytes, they developed neutrophilia and their LECs produced high amounts of G-CSF, indicating that increased G-CSF production and granulopoiesis in STAT5^−/−^ mice does not reflect a compensatory mechanism for a decreased absolute lymphocyte count.

In the present study, we have demonstrated that G-CSF expression in LECs is repressed by cytoplasmic rather than by nuclear STAT5b. It is known that STAT5 can function to not only stimulate but also inhibit gene transcription [33], and a number of mechanisms have been described by which STAT5 acts as a repressor [34], [35]. For example, consistent with our observations, the STAT5b VVVI mutant, which remains primarily cytoplasmic and does not accumulate significantly in the nucleus even after prolactin stimulation, is capable of inhibiting STAT1-mediated activation of the interferon regulatory factor-1 promoter at an extranuclear level [35]. An intriguing question is how cytoplasmic STAT5b can repress G-CSF expression in LECs. Regulation of G-CSF gene expression is achieved at the level of transcription as well as post-transcriptionally [15]. Thus, a possible mechanism may be that STAT5b competes for a cytoplasmic factor required for G-CSF gene transcription in the nucleus. Alternatively, cytoplasmic STAT5b may interfere with RNA-binding proteins or RNAse activity, thereby increasing G-CSF transcript instability. Further studies are needed to address these questions. Enhancement of G-CSF mRNA and protein expression in pro-inflammatory cytokine-stimulated LECs follows rapid degradation of cytoplasmic STAT5b. It has been reported that the adapter molecule c-Cbl negatively regulates STAT5-mediated transcription by inducing ubiquitination and proteosomal degradation of cytoplasmic STAT5 [29]. Similarly, our results show that in LECs, pro-inflammatory cytokines activate intracellular pathways leading to proteosomal degradation of STAT5b.

## Materials and Methods

### Mice

**Figure 8:**
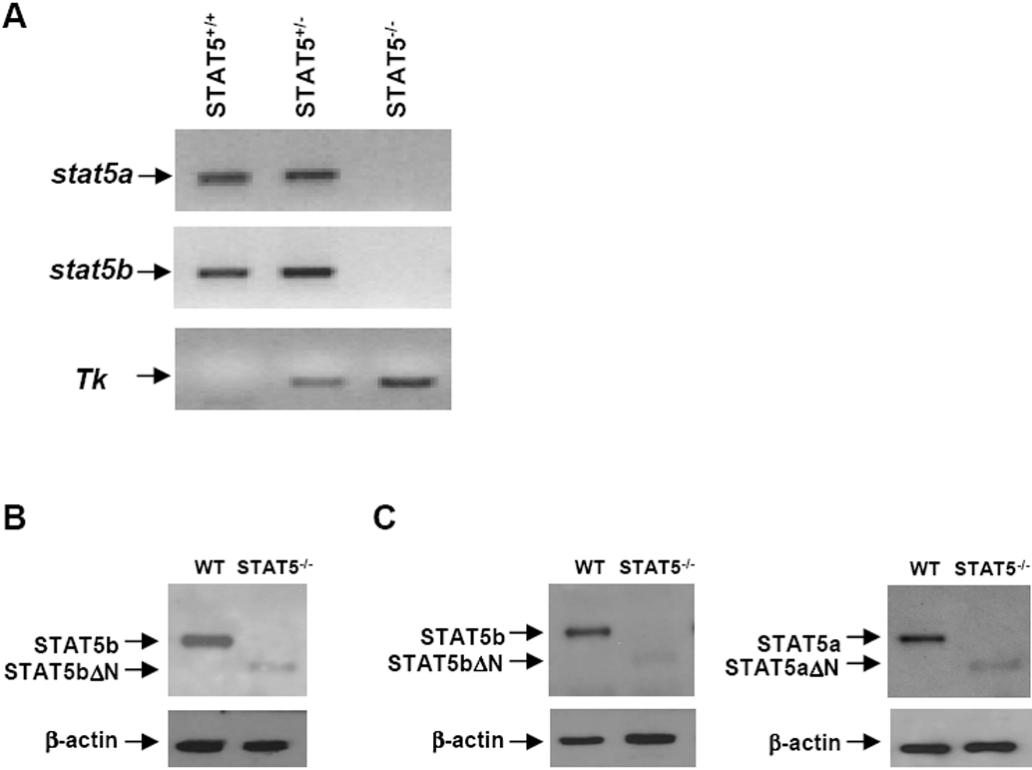
Characterization of wild-type and STAT5^−/−^ C57Bl/6 mice. (A) Tail DNA from STAT5^+/+^, STAT5^+/−^, and STAT5^−/−^ C57Bl/6 mice was prepared and PCR were performed to amplify wild-type *stat5a* and *stat5b* DNA as well as the TK cassette. The primers are given in the Material and methods section. (B) LSECs were isolated from wild-type (WT) and STAT5^−/−^ mice. Whole-cell extracts were prepared and analyzed for STAT5b and STAT5bΔN expression by immunoblot. We used an antibody (G-2) directed to an epitope mapping at the C-terminus of STAT5b. Equal loading of proteins on the gel was confirmed by probing the blots for β-actin. (C) Blood neutrophils were isolated from WT and STAT5^−/−^ mice. Whole-cell extracts were prepared and analyzed for STAT5b/STAT5bΔN (left panel) and STAT5a/STAT5aΔN (right panel) expression by immunoblot. For STAT5b/STAT5bΔN, we used the G-2 antibody. For STAT5a/STAT5aΔN, we used an antibody (L-20) raised against a peptide mapping at the C-terminus of STAT5a. Equal loading of proteins on the gel was confirmed by probing the blots for β-actin.

All animal protocols were approved by the Animal Ethics Committee of the University of Liège. STAT5^−/−^ mice were obtained from Dr James Ihle (St Jude Children's Research Hospital, Memphis, TN) and backcrossed 8 generations onto the C57Bl/6 background. Heterozygote mice were then crossed to yield homozygous double-knockout mice for analysis. All studies were performed on 6- to 10-week mice, and littermates were always used as wild-type controls. Mice were genotyped by PCR of tail DNA as previously described [23]. The primers consisted of STAT5a: F9 primer (5′-AAGGGACAGGAAGAGAGAAGG-3′), R1 primer (5′-CCCATACAACACTTGCATCT-3′); herpes simplex virus thymidine kinase (TK) cassette: TKp primer (5′-GCAAAACCACACTGCTCGAC-3′); STAT5b: R8 primer (5′-GGAGATCTGCTGGCTGAAAG-3′), F11 primer (5′-TCAAACACACCTCAATTAGTC-3′). A representative PCR performed on tail DNA from STAT5^+/+^, STAT5^+/−^, and STAT5^−/−^ mice is provided in Figure 8A.

As previously described in the 129/SvJ background [23], approximately one-third of the double-knockout C57Bl/6 mice died within 48 hr. Moreover, the STAT5a/b-deficient C57Bl/6 mice were smaller than wt counterparts (the STAT5^−/−^ mice weighed ∼30% less than the wt littermates), and the females were infertile, as previously reported by Teglund et al. [23].

It has been demonstrated that N-terminally truncated STAT5a and STAT5b proteins (STAT5aΔN and STAT5bΔN) may be produced in several cell types of STAT5^−/−^ mice [36]. These proteins lack approximately 100 amino acids, but are likely to display some transcriptional activity. Immunoblots were performed in order to ensure that the STAT5ΔN proteins were only expressed at low levels in STAT5^−/−^ neutrophils and LSECs, and therefore to ascertain that our observations in deficient mice were not the result of some aberrant function of the truncated STAT5 proteins. Our experiments showed that the STAT5ΔN proteins were barely detectable in STAT5^−/−^ neutrophils and LSECs (Figures 8B and 8C). STAT5aΔN and STAT5bΔN only occurred at 5% or less of the normal levels, as determined by densitometry.

### Peripheral Blood Counts

Complete blood counts were determined using a Coulter counter. Differentials counts were performed on blood smears stained with Wright-Giemsa.

### Thioglycollate-Induced Peritonitis

Mice were injected intraperitoneally with 2 ml of 3% TGA broth (DIFCO). Peritoneal exudate cells were harvested after 4 h by PBS lavage. Red blood cells were lysed in Tris-buffered ammonium chloride (pH 7.2) buffer, and leukocytes were enumerated using a Coulter counter. Leukocyte differentials performed on cytospins stained with Wright-Giemsa showed that >95% of the peritoneal exudate cells were neutrophils. Peritoneal cells were also stained with anti-CD11b-FITC and anti-Gr-1-PE antibodies (BD Pharmingen), and analyzed by FACS.

### Respiratory Burst Assay

Dihydrorhodamine 123 oxidation assay was performed as described [37]. In brief, 1×10^6^ peritoneal neutrophils harvested by lavage from TGA-treated mice were incubated with 100 µM dihydrorhodamine and 1000 U/ml catalase in 100 µL of medium at 37°C for 5 min. One hundred microliters of medium containing 200 ng PMA was added and incubated for another 20 min. Cells were washed with cold PBS twice, resuspended in PBS containing 1% paraformaldehyde, and analyzed by FACS.

### Phagocytosis and Bactericidal Assays

Phagocytosis and bactericidal assays were performed as described [21] with some modifications. In brief, peritoneal neutrophils from TGA-treated mice were washed and resuspended in medium lacking antibiotics. 10^7^ live *Staphylococcus aureus* labeled with 5 µM CFSE was mixed with 2×10^6^ neutrophils and incubated at 37°C for 30 min. Subsequently, extracellular bacteria were killed by incubation for an additional 30 min with 50 µg/ml gentamycin. Phagocytic activity was assessed by FACS. To measure bactericidal activity, engulfed bacteria were released by lysing neutrophils in 1 ml water. One hundred microliters of a 1∶1,000 dilution of the bacterial suspension was plated and colonies were counted after a 24 h incubation at 37°C.

### Bone Marrow Cytology

Bone marrow cells were collected from both hind limbs (tibias and femurs), counted using a Coulter counter, prepared by cytospin centrifugation, and stained with Wright-Giemsa. Differential cell counts were scored visually on coded slides. Mature marrow neutrophils were counted based on CD11b and Gr-1 expression (CD11b^+^Gr-1^hi^ cells). The bone marrow precursor populations were counted on the basis of the expression of specific membrane markers [38]. The CMP population was identified as Lin^−^Sca-1^−^IL-7Rα^−^c-kit^+^CD34^+^FcγR^low^ cells. GMPs were defined as Lin^−^Sca-1^−^IL-7Rα^−^c-kit^+^CD34^+^FcγR^+^ cells. HSCs were defined as Lin^−^Sca-1^hi^IL-7Rα^−^c-kit^hi^.

### Isolation of Mature Neutrophils and Bone Marrow Progenitors

Blood was collected into heparinized tubes. Red blood cells were removed by lysis in hypotonic buffer. Cells were then washed twice in PBS, stained with biotin-conjugated anti-Gr-1 antibody (BD Pharmingen), and selected with streptavidin-coated magnetic beads (Miltenyi Biotech). Marrow neutrophils were FACS-sorted based on CD11b and Gr-1 expression (CD11b^+^Gr-1^hi^ cells). Purity of isolated cells, as determined by counting of cytospin preparations stained with Wright-Giemsa, was always >90%. HSCs, CMPs, and GMPs were FACS-sorted based on the expression of specific membrane markers (see above). Neutrophils were cultured at 37°C in RPMI 1640 medium supplemented with 1% glutamine, 10% FCS, 50 µg/ml streptomycin, and 50 IU/ml penicillin.

### Apoptosis Assays

Neutrophils were assayed for apoptosis using dual color annexin-V-FITC/propidium iodide staining (Roche) and flow cytometry analyses. Freshly isolated progenitors were assayed for apoptosis using trypan blue exclusion. For antiapoptosis assays, neutrophils were cultured for 24 h in the presence of 0.5 or 25 ng/ml G-CSF or 25 ng/ml GM-CSF.

### Proliferation Assays

Bone marrow cells were seeded at 2×10^5^ cells per well and incubated for 48 h at 37°C in the presence of increasing concentrations of recombinant murine G-CSF. Cells were then pulsed with 1 µCi ^3^[H]thymidine and incubated for a further 16 h prior to harvest.

### Real Time Quantitative RT-PCR

Total RNA from various organs and tissues or from stimulated LSECs was extracted using Trizol (Invitrogen) and reverse transcribed with the AMV reverse transcriptase (Roche). Amplification reactions were performed using SybrGreen reaction mix (Eurogentec) in the presence of 0.5 µl of total cDNA and 300 nM of specific primers for either murine G-CSF (TGCTTAAGTCCCTGGAGCAA and AGCTTGTAGGTGGCACACAA) or murine STAT5b (CAGGTGGTCCCCGAGTTTGCA and CAGATCGAAGTCCCCATCGGTA). Real time PCR and fluorescence quantification were performed in a Lightcycler GenAmp 5700 (Applied Biosystems). The level of β-actin mRNA was used as an internal control for normalization.

### Immunohistochemistry

Immunohistochemical techniques used in this study were described in detail by Pajak et al [39]. Organs were fixed for 3 days in Immunohistofix (A PHASE) followed by dehydration in a graded series of ethanol (30, 50, 70, 90, and 100%) for 30 min each at room temperature. Tissues were embedded in Immunohistowax (A PHASE), sectioned at 3 to 6 µm, de-embedded by washing in acetone for 5 min, and transferred to PBS. The endogenous peroxidase activity was neutralized by 3% H_2_O_2_ in PBS for 60 min. Tissue sections were treated for 30 min with 1% blocking reagent (BR; Boehringer), and the slides were incubated with 10 µg/ml anti-G-CSF rabbit polyclonal antibody (FL-207, Santa Cruz Biotechnology). Slides were then incubated with 20 µg/ml protein A-HRP (Sigma) and stained with a solution of 3-amino-9-ethylcarbazole (Sigma). Sections were counterstained with Mayer's hemalum (Merck). No staining was observed when the primary antibody was omitted.

### Immunofluorescence Confocal Microscopy

Livers were frozen in isopentane and 10 µm cryostat sections were prepared. Sections were air dried and saturated in PBS plus 1% BR mixed with equal volume of culture supernatant containing anti-mouse CD16/CD32 monoclonal antibodies (2.4G2). Slides were washed in PBS, and incubated for 1 h at room temperature with 25 µg/ml anti-STAT5b mouse monoclonal antibody (G-2; Santa Cruz Bitoechnology) in PBS-BR-2.4G2. The slides where then washed three times with PBS-BR and incubated for 1 h with a 1∶200 dilution of Alexa-488-coupled goat anti-mouse IgG (Molecular Probe) and with 20 µM hexidium iodide (Molecular Probe) for nuclear staining. The samples were then washed three times in PBS containing 0.2% gelatine and mounted for analysis on a Zeiss LSM510 laser scanning confocal microscope. No staining was observed when the primary antibody was omitted. No signal was observed when the slides were stained using an anti-STAT5a antibody (L-20; Santa Cruz Biotechnology).

### Isolation of Liver Sinusoidal Endothelial Cells

LSECs were isolated as previously described [40], seeded onto 6-well flat-bottom plates (Becton Dickinson) at a density of 5×10^6^ cells/well, and kept in culture for 3 days before experiments were performed. In some experiments, LSECs were stimulated with 100 IU/ml recombinant murine TNF-α and 500 pg/ml recombinant murine IL-1β (Peprotech).

### Inflammatory Stimulation

To induce inflammation, mice were injected intravenously with 2 µg recombinant murine TNF-α and 2 µg recombinant murine IL-1β (Peprotech).

### Immunoblots

Cytoplasmic, nuclear and whole-cell extracts as well as immunoblots were performed as previously described [41]. Various anti-STAT5a and/or anti-STAT5b antibodies were used (G-2, L-20, N-20, C-17; Santa Cruz Biotechnology). An anti-STAT5b antibody (G-2) was used in Figures 5D, 5G, 8B, and 8C. STAT5b G-2 is a mouse monoclonal antibody epitope mapping at the C-terminus of STAT5b of mouse origin. An anti-STAT5a antibody (L-20) was used in Figure 8C. STAT5a L-20 is an affinity purified rabbit polyclonal antibody raised against a peptide mapping at the C-terminus of STAT5a of mouse origin. Equal loading of proteins on the gel was confirmed by probing the blots for β-actin or Oct-1. In experiments aimed at determining whether STAT5b was degraded by the proteasome, LSECs were treated with 20 µM MG132 (AG Scientific Inc.).

### Electrophoretic Mobility Shift Assays

Electrophoretic mobility shift assays and supershift experiments were performed as described elsewhere [39]. The sequence of the STAT5 probe (viz. the 21 bp GAS-like element from the promoter of the bovine β-casein) used in this work was as follows: 5′-AGATTTCTAGGAATTCAAATC-3′. To confirm specificity, competition assays were performed with a 50-fold excess of unlabeled wild-type probes and with mutated probes (data not shown). Binding of the non inducible transcription factor Oct-1 was used as an internal standard. In some experiments, nuclear extracts from MAC-T cells (bovine mammary epithelial cells; NEXIA Biotechnologies Inc.) stimulated with purified bovine prolactin were used as positive controls for STAT5 activation.

### Cytokine Assays

G-CSF, GM-CSF, IL-3, SCF, and TNF-α protein levels in serum and/or in culture supernatants were determined by ELISA (R&D or Endogen).

### Bone Marrow Transplantation

5×10^6^ wild-type or STAT5^−/−^ bone marrow cells were injected intravenously into lethally irradiated (1000 rad) control or mutant mice (8-week-old mice were used). Eight weeks later, blood, bone marrow and liver samples were prepared for Wright-Giemsa staining, FACS analysis, cytokine assays, or immunohistochemistry. There were 6 mice in each experimental group (WT→WT, WT→KO, KO→KO, and KO→WT). The experiments were repeated 3 times.

### Statistical Analysis

Data are presented as means±SDs. The differences between mean values were estimated using either an ANOVA with subsequent Fisher's protected least significant difference tests or a Student's t test for unpaired data. A value of P<0.05 was considered significant. All the experiments were repeated at least three times. n ≥ 6 in each experimental group.
